# Karyotype features of trematode *Himasthla elongata*

**DOI:** 10.1186/s13039-016-0246-8

**Published:** 2016-04-29

**Authors:** Anna I. Solovyeva, Vera N. Stefanova, Olga I. Podgornaya, Serghei Iu. Demin

**Affiliations:** Institute of Cytology RAS, St. Petersburg, 194064 Russia; Saint Petersburg State University, St. Petersburg, 199034 Russia; Far Eastern Federal University, Vladivostok, 690922 Russia

**Keywords:** *Himasthla elongata*, Digenea, Karyotype, 18S rDNA mapping

## Abstract

**Background:**

Trematodes have a complex life cycle with animal host changes and alternation of parthenogenetic and hermaphrodite generations. The parthenogenetic generation of the worm (rediae) from the first intermediate host *Littorina littorea* was used for chromosome spreads production. Karyotype description of parasitic flatworm *Himasthla elongata* Mehlis, 1831 (Digenea: Himasthlidae) based on fluorochrome banding and 18S rDNA mapping.

**Results:**

Chromosome spreads were obtained from cercariae embryos and redial tissue suspensions with high pressure squash method.74.4 % of the analysed spreads contained 12 chromosome pairs (2n = 24). Chromosome classification was performed according to the morphometry and nomenclature published. *H. elongata* spread chromosomes had a rather bead-like structure. Ideograms of DAPI-banded chromosomes contained 130 individual bands. According to flow cytometry data, the *H. elongata* genome contains 1.25 pg of DNA, so one band contains, on average, 9.4 Mb of DNA. Image bank captures of individual high-resolution DAPI-banded chromosomes were provided. Differential DAPI- and CMA_3_-staining revealed the chromatin areas that differed in AT- or GC-content. Both dyes stained chromosomes all along but with varying intensities in different areas. FISH revealed that vast majority (95.0 %) of interphase nuclei contained one signal for 18S rDNA. This corresponded to the number of nucleoli per cell detected by observations *in vivo*. The rDNA signal was observed on one or two homologs of chromosome 10 in 72.2 % of analysed chromosome spreads, therefore chromosome 10 possessed the main rDNA cluster and minor ones on chromosomes 3 and 6, that corresponds with AgNOR results.

**Conclusions:**

*Himasthla elongata* chromosomes variations presented as image bank. Differential chromosome staining with fluorochromes and FISH used for 18S rDNA mapping let us to conclude: (1) *Himasthla elongata* karyotype is 2n = 24; (2) chromosome number deviates from the previously studied echinostomatids (2n = 14–22); (3). Chromosome 10 possesses the main rDNA cluster with the minor ones existing on chromosomes 3 and 6.

**Electronic supplementary material:**

The online version of this article (doi:10.1186/s13039-016-0246-8) contains supplementary material, which is available to authorized users.

## Background

The digenetic trematodes, or flukes, are ones of the most common and abundant of parasitic worms. They act as parasites on all classes of vertebrates, especially marine fishes, and nearly every organ of the vertebrate body can be parasitised by some kind of trematode, adult or juvenile [1]. Many trematode species are the causative agents for massive zoonosises. The list of flukes infectious to humans is quite large, and because of their importance, numerous investigations have been initiated, especially regarding parasite-host interactions [1]. Human parasites as model objects require appropriate laboratory conditions. The subclass Digenea comprises about 18000 species and it is possible to find a safe alternative for parasite research. Genus *Himasthla* is an example of a safe research option. There are 25 presently described species of *Himasthla Mehlis,* 1831 (Digenea: Himasthlidae [2]). Just two of them were found in fishes and one in humans; all three cases seemed to be accidental infections [3]. However, most are studied quite insufficiently, excepting *Himasthla elongata* (Mehlis, 1831), which became a new model for ecological, immunologic and molecular investigations [4–7].

*H. elongata* is common in the coastal ecosystems of northern European seas. Like other trematodes, it has a complex lifecycle dependent on of host and parthenogenetic (redia, cercaria) and hermaphrodite generations. The first intermediate hosts of this parasite are intertidal snails of the *Littorina* (Gastropoda, Prosobranchia) genus and the second intermediate hosts are the intertidal bivalves, *Mytilus edulis* and *Cerastoderma edule*; gulls are its final hosts [8].

Trematode metacercariae parasitising bivalve molluscs may influence the vital functions of the hosts, lowering their resistance to unfavourable environmental factors and, in the case of intensive infection, even causing their death and resulting in mass mollusc mortality [4].

Cytogenetic studies of parasites are useful not only for understanding systematics, but also the basic mechanisms underlying parasitic agents. Among invertebrates, chromosome mapping has been carried out for a few model organisms due to methodological difficulties.

*H. elongata* becomes a model for zoological and molecular studies, but it has not been studied at the cytogenetic level. The lack of knowledge about karyotype makes problems for genomic investigation. Moreover, propelled by ever-increasing throughput and decreasing costs, next generation sequencing (NGS) has produced a growing number of genomes and transcriptomes sequenced (existing in databases) and it looks like the *H. elongata* genome will be sequenced soon. Newly sequenced genome should be assembled and attached to a chromosome’s physical map, thus it is necessary to acquire the data on its karyotype. The purpose of the current study is the description of *H. elongata* chromosomes. DNA sequence mapping is most convenient for carrying out counterstaining chromosome bands with fluorochromes. DAPI excitation emission varies in proportion to the AT-content of DNA and chromatin condensation level [9]. We elaborated protocols for chromosome preparation and fluorescence *in situ* hybridisation (FISH), enabling conservation of the typical pattern of DAPI-banding for all components of the karyotype. To establish the position of GC-rich bands on individual chromosome sets, a GC-specific fluorochrome, chromomycin A_3_ (CMA_3_), was used for staining in addition to DAPI counterstaining. The present study is focused on karyotype description of the parasitic flatworm, *H. elongata*, based on fluorochrome banding and 18S rDNA mapping.

## Methods

### Sampling site and collection of parasites

A collection of periwinkles infected with *H. elongata* was obtained from, along with cell suspension preparation carried out at the White Sea Biological Station of the Zoological Institute of the Russian Academy of Sciences situated in the Chupa Inlet, the Kandalaksha Bay of the White Sea (66°20′N; 33°38′E) during July and early August in 2012 and 2013. Digeneans were identified on the basis of their mollusc hosts and their morphological [3] and molecular (18S rDNA) features. At least 8 snails with only *H. elongata* infection i.e. 8 populations of *H. elongata* parthenogenetic larvae obtained from their hosts were used in cytogenetic experiments.

### Intravital observations

Live materials were observed under a Leika DM2500 microscope. Dark field illumination at low magnification (objective 10×) was realised by use of the Ph3 phase diaphragm in combination with closed differential interference contrast (DIC) prisms. DIC was utilized for observations under the 100× DIC objective.

### Metaphase chromosome spread preparation

*H. elongata* parthenitae - rediae were obtained in amount of several hundreds individuals from each *L. littorea* snails and washed from the host tissue by three exchanges of seawater filtered through a 0.22 mm Millipore filter. The worms were incubated in Leiboviz L-15 (Sigma) medium with 0,01 mg/ml gentamycin (PanECO, Russia) and 0.1 % colchicine (PanECO, Russia) for 4 h at room temperature and treated with hypotonic solution (5 mM KCl) for 40 min, then fixed with Carnoy’s solution (methanol:glacial acetic acid mixture; 3:1). Fixed rediae were repeatedly passed through the syringe with a 22 G needle. The suspension was placed in 15 ml tubes and kept still for 3–5 min to sediment large fragments. The top phase was collected and centrifuged three times at 2.5 krpm for 10 min with the three changes of fixative and stored at −20 °C until slide preparation. Chromosome spreads were prepared according to classic cytogenetical protocols used for trematodes [10–15] with convenient air-drying method, along with more recent techniques, such as high-pressure squash preparation [16, 17]. The convenient air-drying method was performed as follows: 4 or 5 drops of cell suspension were carefully placed onto slides which had been previously chilled in ice water for maintaining a thin film of water at the time when the drops fall on the slide from a height of about 20 cm. Slides were air-dried and then stored at −20 °C until staining. A modified protocol from Deng et al. [14] was performed the next way: the washed slides were placed an a stainless steel bar inside a moist chamber. 30 μl of cell suspension were dropped on each slide Then the moist chamber was at 50 °C in thermostat until fixative drying.

### High-pressure squash preparation

Whole *H. elongata* rediae were fixed after colchicine and hypotonic solution treatment and used for spreading chromosomes. On average 50 rediae from different snails were used for slides preparations. The suspension of dissociated into small pieces worms’ tissues was dropped on slides containing Carnoy’s solution on the surface. The spread cells were coated with a 50.0 % propionic acid drop and then covered by 24 × 24 mm cover slips immediately after fixative evaporation. A mechanical vise was used to evenly apply pressure to further flatten chromosomes on the preparation. Approximately 150 kg/cm^2^ of pressure through the precision vise was gradually applied during 90–120 second intervals. At that point, the slides were placed into liquid nitrogen and the cover slips were removed. Afterwards, the slides were dehydrated in a series of ethanol (70.0, 80.0, and 100.0 %), air-dried and kept in −20 °C until staining.

### Giemsa staining

The slides were stained in a 3.0 % solution of Giemsa dye (Merck, USA) in phosphate buffer solution (pH 6.8) for 12 min and flushed with flowing water.

### Fluorescent *in situ* hybridization with 18S rDNA probe

As the *H. elongata* genome is not yet sequenced and 18S rDNA is quite conservative, a small subunit ribosomal probe was generated by polimerase chain reaction (PCR) using the 18Sa forward primer (AACCTGGTTGATCCTGCCA) and the 18Sb reverse primer (GATCCTTCTGCAGGTTCACCTAC) [18]. The PCR product was sequenced in order to confirm its attribution to 18S rDNA and submitted to GenBank (KU886143). The analysis of a 18S rDNA probe sequence was performed with the BLAST tool [19]. Isolation of genomic DNA was performed according to Winnenpeninx [20]. The probes were labelled with biotin-14-dUTP under appropriate conditions. Slide pretreatment was performed according to Khodyuchenko et al. [21] with modifications: chromosome preparations were digested with 100 μg/ml RNase A in 2 × SSC for 1 h at 37 °C and washed twice in 2 × SSC for 5 min each, then prefixed with 2.0 % PFA for 15 min, and then washed with 1 × PBS three times for 5 min at a time and incubated in 0.1 % Triton X-100 for 10 min and washed again with 1 × PBS. Hybridisation at 37 °C for 18 h was followed by the washes, which included 0.2 × SSC (3 × 5 min, 60 °C) and 2 × SSC (3 × 5 min, 42 °C). Probe signals were detected with streptavidin – Alexa Fluor 594 conjugate (Life technologies, USA) in blocking solution. The slides were counterstained with Slow Fade Gold Antifade with DAPI (Molecular Probes, USA).

### Double Chromomycin A_3_ - DAPI staining

The slides were stained with Chromomycin A_3_ (CMA_3_) based on Schweizer [22] with several modifications. The stock solution of chromomycin A_3_ (Sigma-Aldrich) (1 mg/ml) was prepared by dissolution in deionised water without stirring for several days at 4 °C in the dark. Older solutions tend to stain better. Working solution of CMA_3_ (0.5 mg/ml) was prepared by dissolving (1:1) the stock solution in Mcllvaine’s buffer (pH = 7.0) with 5 mM MgCl_2_. Slides were rinsed in McIlvaine’s buffer and placed in CMA_3_ working solution under a coverslip and stained in the dark at RT for 1 to 2 hours. To remove the coverslip, the slides were briefly washed in McIlvaine’s buffer and air-dried. After that a few drops of DAPI solution are placed on slides and covered with a coverslips. Slides are stained in the dark for 20–30 min at RT, rinsed in McIlvaine’s buffer and air-dried. DAPI stock solution (1 mg/ml) is prepared on deionized water and can be stored frozen in dark for a year. DAPI working solution (0.8mkg/ml) is prepared on McIlvaine’s buffer (pH = 7.0) usually fresh before use. Then the slides were mounted in ProLongR Gold antifade (Invitrogen) and sealed with nail polish. Stained slides were aged for 3 to 5 days in the dark at 4 °C to stabilise CMA_3_ fluorescence before examination.

### Fluorescence microscopy

Chromosome spreads were examined with a Leica Fluorescence Microscope DMI 6000B (Leica Wetzlar GmbH, Germany) at the Development of Molecular and Cellular Technologies Resource Centre at Saint-Petersburg State University. Images were taken with a 100×/1.4 oil immersion objective using appropriate filter cubes fluorescent dyes, like CMA_3_(430–480 nm), Alexa 594 and DAPI(360–390 nm), and recorded using a monochrome-cooled CCD camera. Karyological data of *H. elongata* (relative length and centromeric index) were calculated in 64 best spreads out of 100 evaluated spreads with Image Tool 3.0 software [23]. The centromere position on the chromosomes was classified according to the nomenclature of Levan et al. [24]. Negative images of DAPI-stained chromosomes were enhanced in Adobe Photoshop version 4 as described before [25].

## Results

**Fig. 1:**
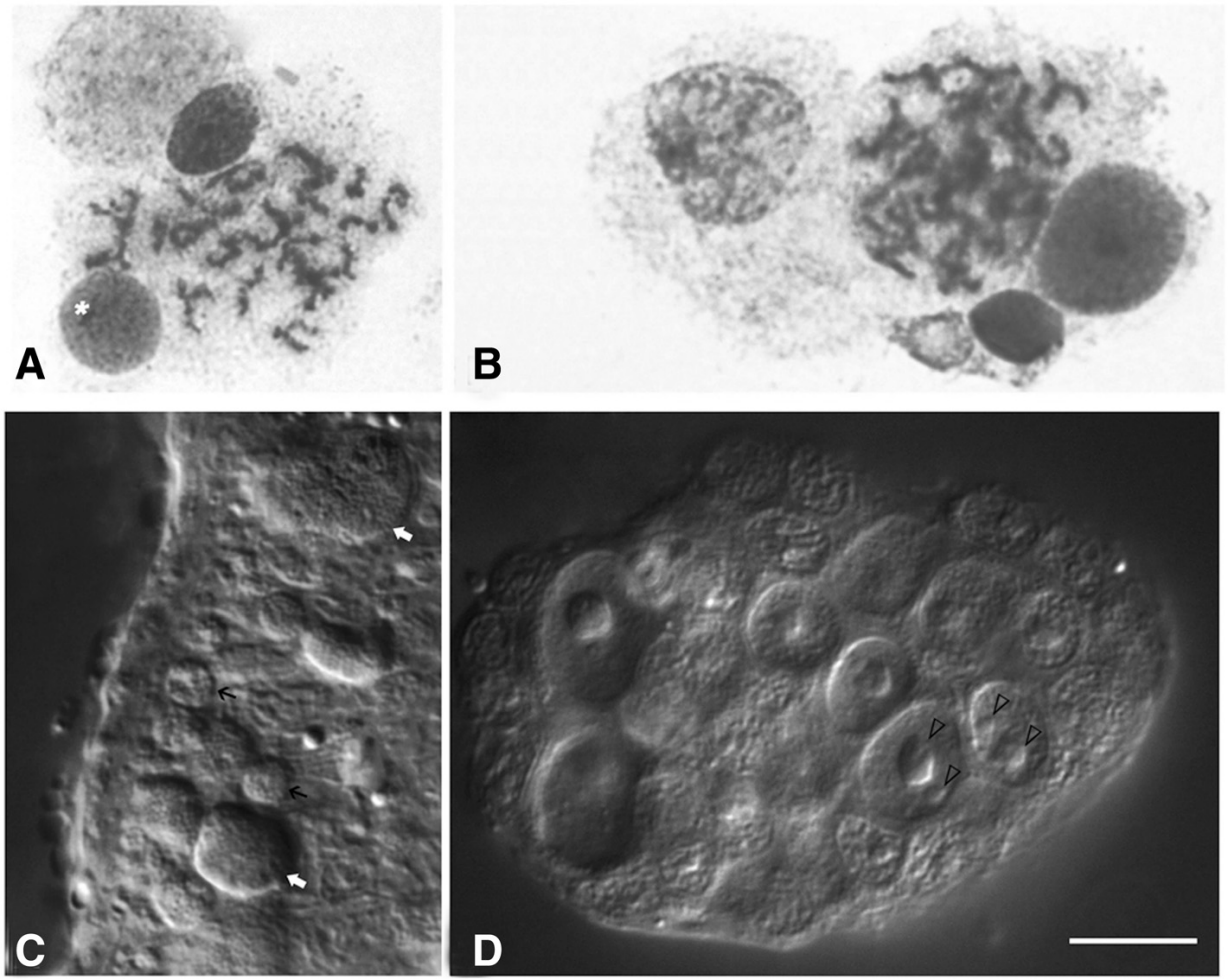
**a**, **b** flattened small pieces of tissues with large sized prometaphase-metaphase and interphase cells in association with small sized, probably senescent or stem cells (asterisk), dissociated mature rediae (Giemsa staining). **c**, **d** DIC images of alive juvenile cercaria (lateral fragment of the body) and its embryo; (**c**) – tegumental margin with large subtegumental glandular cells (white arrows) and small, probably senescent or stem cells (black arrows); (**d**) cercarial embryo, nucleoli are visible (arrowheads). Scale bar – 10 μm

**Fig. 2:**
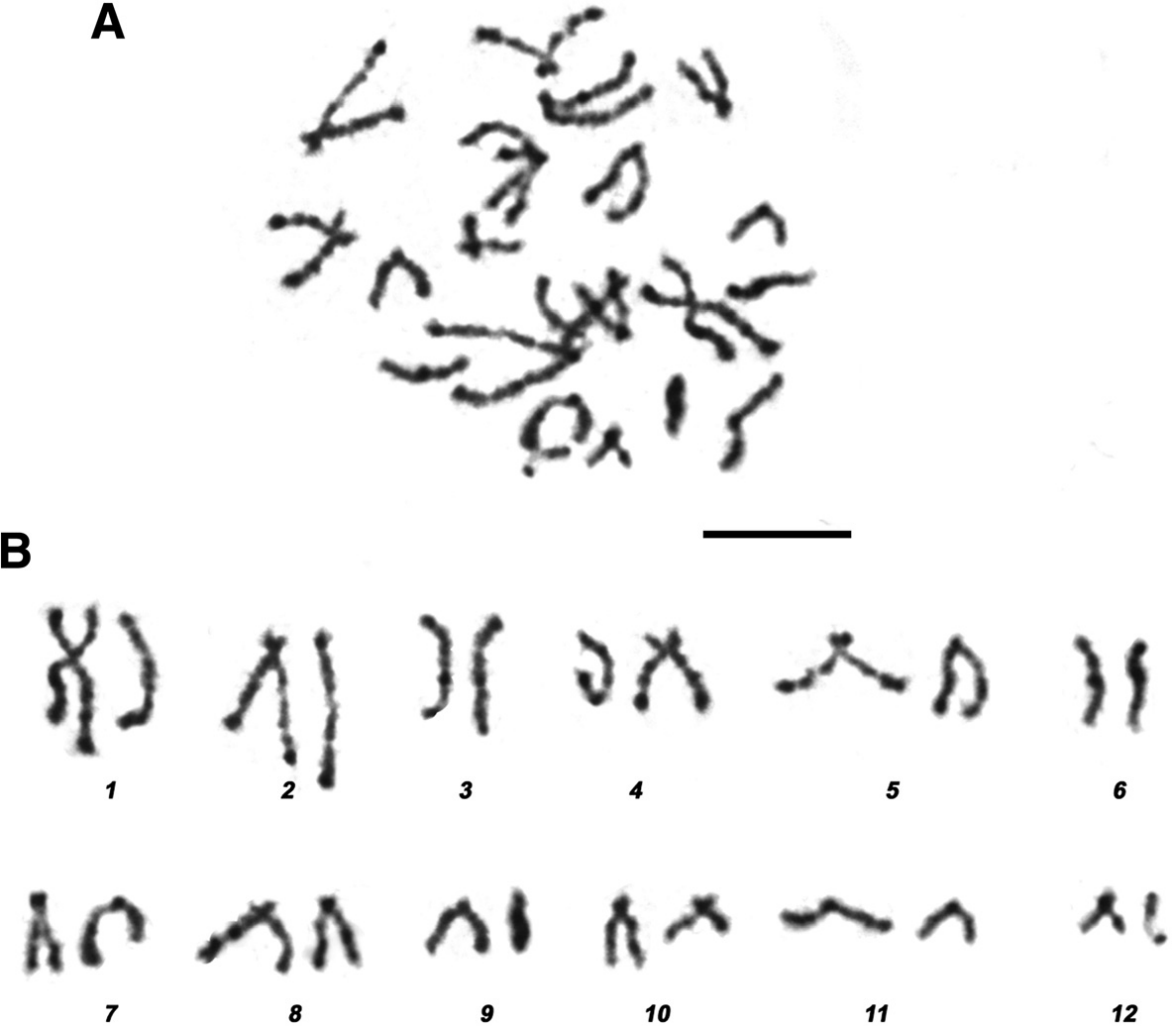
**a** *H.elongata* DAPI-stained chromosome spreads in grey scale. **b** karyotype shown in **a**. Scale bar – 10 μm

Identification of a prometaphase and metaphase chromosome source among larval cells was carried out by comparing cell morphology at preparations of shredded and fixed rediae tissues with preparations of live juvenile cercariae or embryos at different developmental stages. Cytological preparations stained with Giemsa contained cells of various sizes and morphology (Fig. 1a, b). All slides contained large amounts of resting cells, which contained a round or oval 6–8 μm diameter nuclei, uniformly filled with condensed chromatin, and possessed a narrow cytoplasm rim with a width of 1 μm. The vast majority of chromosome spreads were determined among the large round or oval cells with a 10–20 μm core diameter, representing no more than 1.0–5.0 % of all cells in the preparation. These cells had either an acentric core and a developed cytoplasm or a centrally located nucleus and a narrow (no wider than 3 μm) layer of cytoplasm (Fig. 1c, d). According to histological and cytological features, the large cells located near the tegument may correspond to subtegumental glandular cells – socalled cyton precursors, − the tegument nucleus-bearing compound [26]. This source was used for chromosome preparations. The prometaphase spreads were obtained with a high pressure (~150 kg/cm^2^) spreading technique [16] applied to the cell suspension, made from shredded rediae and cercariae embryos. Compared with a convenient air-drying method and a complexed protocol described by Deng et al. [14], the chromosomes, treated with pressure had a much better bands resolution, therefore we considered to call them “high-resolution” chromosomes. An example of a metaphase chromosomes’ DAPI-banding pattern is shown in Fig. 2.

About 74.4 % of the spreads analysed contained 12 chromosome pairs (2n = 24), while the others were aneuploid. Generally, *H. elongata* chromosomes had a rather bead-like structure than a banded one. Prometaphase-metaphase chromosomes of *H. elongata* large sized cells relatively rare had conjugated sister chromatids (SCs). Usually their SCs were dissociated elsewhere for the exception of centromeric region. X- or Λ-shaped chromosome figures that is considered typical only for metaphase, formed as the result. Sister chromatids were associated only in the centromere region in approximately half of the typical prometaphase-metaphase chromosomes with dissociated SCs. In the other half, they had a conjugated SCs yielding to a rod-shaped or I-shaped form. Chromomeric patterns of sister chromatids for the chromosomes with dissociated SCs were similar but not the same. Such chromosomes consisted of two 0.5–2 μm collaterally-associated chromatin strands with clearly visible primary (centromeric) constriction. Such primary constriction was observed quite rarely in chromosomes with conjugated SCs. The frequencies chromosomes with different type of SCs association observed in prometaphase-metaphase spreads are summarised in Table 1.

**Table 1:** Frequencies of chromosomes with dissociated sister chromatids in *H. elongata* chromosome spreads (*N* = 100)

Number of chromosomes with dissociated SCs in each spread	4	5	6	7	8	9	10	11	12	13	14	15	16	17	18	19	20	21	23
% of spreads with chromosomes with dissociated SCs	1.0	3.0	1.0	2.0	4.0	11.0	7.0	13.0	6.0	9.0	8.0	9.0	10.0	3.0	4.0	3.0	3.0	2.0	1.0

The top row of the table indicates the number of chromosomes with dissociated sister chromatids (SCs) detected in each spread. The bottom row of the table indicates the percent of corresponding cells among 100 spreads analysed

The difference in chromosome shapes may reflect the dynamics of sister chromatid segregation during cell division (Table 1). Not a single spread exhibited complete segregation, i.e. in all the set (24) with 2 chromatids, usually about half of the set already went through segregation. Metaphase DAPI-banded karyotype of *H. elongata* (2n = 24, Fig. 2) allows chromosomes’ classification. Table 2 demonstrates the morphometric data for each set of chromosomes. All measurements and centromeric index calculations were performed for metaphase chromosomes with dissociated SCs. Pairs 2, 4–7 and 11 and 12 can accurately be classified as subtelocentric, pair 3 – metacentric. The classification of chromosomes 1 (m-sm) and 8–10 (sm-st) is uncertain for centromeric index values SD is on the border of two types [24].

**Table 2:** Relative length (means ± SD) and centromeric index of *H. elongata* chromosomes

Chromosome №	Relative length, %	Centromeric index, %	Classification
1	14.6 ± 1.7	37.2 ± 2.8	m-sm
2	12.6 ± 2.1	14.9 ± 2.3	st
3	10.8 ± 2.0	42.2 ± 3.8	m
4	9.4 ± 1.4	20.2 ± 3.1	st
5	8.4 ± 1.5	21.4 ± 2.9	st
6	8.3 ± 1.6	15.8 ± 2.6	st
7	8.2 ± 1.4	14.8 ± 2.8	st
8	7.0 ± 1.1	24.2 ± 3.4	sm-st
9	6.5 ± 1.0	24.5 ± 2.9	sm-st
10	5.6 ± 1.0	27.1 ± 3.6	sm-st
11	5.2 ± 0.9	20.2 ± 2.8	st
12	4.1 ± 1.9	20.7 ± 2.3	st

*m* metacentric, *sm* submetacentric, *st* subtelocentric (= acrocentric)

**Fig. 3:**
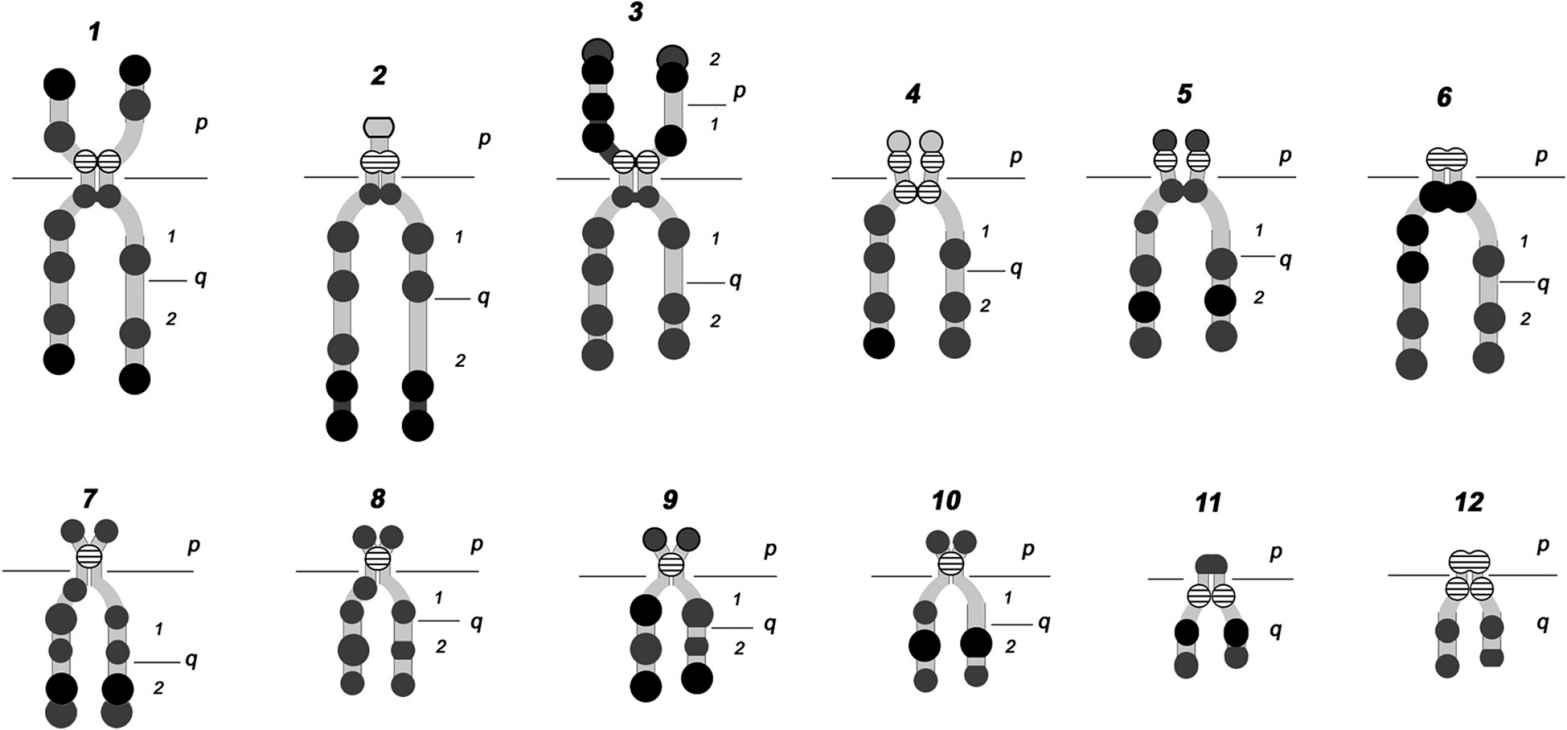
*H. elongata* chromosomes ideogram. Left chromatid on each ideogram represents the summary of chromosome bands with the best resolution and maximum amount of bands visible; the right chromatid represent chromatid often observed; p and q arms are marked. Differences between sizes of DAPI-bands are indicated by circles of two sizes, fluorescence intensity are marked by three shades in grey scale. The terminal bands (chromosomes 2–5, 9) which are designated as black line bordered circles, are not always detected after DAPI-staining. Heterochromatin containing bands are designated with hatching. The straight lines adjacent to the chromosome on both sides indicate the centromere position

High-resolution DAPI-banded *H. elongata* chromosome ideogram construction was based on the results of relative chromosome length and the centromeric index counted (Table 2) as well as morphology. Graphic ideograms were based on the negative image of DAPI-banded chromosomes with maximum bands resolution. *H. elongata* chromosomes possess a typical chromomere (necklace-like) structure. Such morphology is characteristic of human and animal pachytene meiotic chromosomes, but not mitotic chromosomes [27, 28]. All chromosomes contained a block of centromeric heterochromatin. Homologous chromosomes within a single cell could possess centromeric heterochromatin of different sizes; they also vary at different metaphase plates. Ideograms of *H. elongata* high-resolution DAPI-banded chromosomes contain 130 individual bands resolved in haploid chromosomes set (Fig. 3). According to flow cytometry data, the *H. elongata* genome contains 1.25 pg of DNA [29] (for a detailed information see Additional file 1: Figure S1 and Additional file 2: Supplementary methods). Simple recalculation (1 pg DNA = 978 Mb) [30] shows that one band in *H. elongata* chromosome contains, on average 9.4 Mb of DNA.

**Fig. 4:**
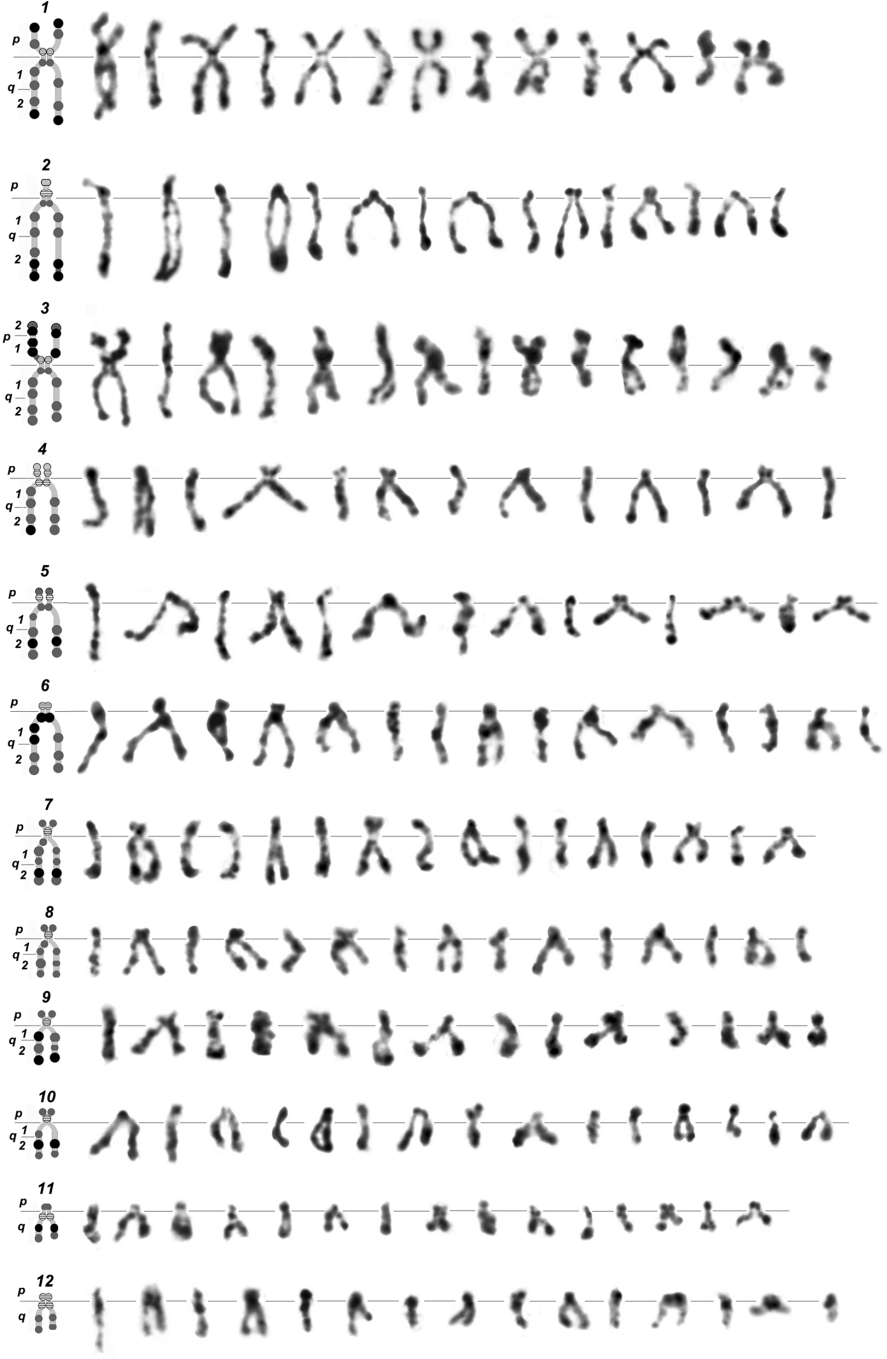
Representative rows of individual DAPI-banded *H. elongata* chromosomes. Each row begins with the ideogram of chromosome ; p and q arms marked; Differences between sizes of DAPI-bands are indicated by circles of two sizes, fluorescence intensity are marked by three shades in grey scale. The following row shows the main chromosome structural variations. Chromosomes are arranged according to decrease of their length and bands’ resolution. The straight lines adjacent to the chromosome on both sides indicate the centromere position

The image bank of individual DAPI-banded *H. elongata* chromosomes consists of 12 chromosome rows: each row represents one of the 12 chromosomes of the set (Fig. 4). Each chromosome row shows the basic structural variations typical for the similar sets. Chromosomes are arranged in row according to decreasing length and, consequently, band visibility (i.e. they have morphological differences). Each row shows all two types of chromosome morphology: chromosomes with conjugated or dissociated SCs. Sister chromatid heteromorphism is related to secondary constrictions of chromosomes with dissociated SCs. Nomenclature used for the description of the individual bands is traditional for animal cytogenetics [31]. Chromatid heteromorphism as a consequence of chromomere difference is displayed on the ideograms as circles (Fig. 3, legend). The banding pattern of chromosomes with conjugated SCs corresponds to those of one or another dissociated chromatids.

Sister chromatid variability was observed in the majority of analysed chromosome sets. Interchromosomal variability of premature sister chromatid segregation, leading to the appearance of chromosomes with dissociated SCs, can be characterised by data from 100 randomly selected spreads (Table 3).

**Table 3:** Distribution of different types of homologous sister chromatid segregation in *H. elongata* chromosomes (100 spreads analysed)

Chromosome №	Homologous chromosomes combinations		
	Dissociated SCs	Dissociated and conjugated SCs	Conjugated SCs
1	15.0	45.0	40.0
2	17.0	47.0	36.0
3	13.0	30.0	57.0
4	59.0	26.0	15.0
5	53.0	30.0	17.0
6	45.0	32.0	33.0
7	16.0	35.0	49.0
8	56.0	33.0	11.0
9	66.0	21.0	12.0
10	74.0	20.0	6.0
11	58.0	23.0	19.0
12	10.0	17.0	73.0

Columns represent % of spreads with amount of homologs dissociated or conjugated for correspondent chromosomes

The chromosome pairs are distinguished by different types of sister chromatid segregation patterns: e.g. pairs 4, 5, and 8–11 are represented mostly in form with dissociated SCs, while some pairs are represented mostly by forms with conjugated SCs – pairs 3, 7, and 12. It appears that chromosomes prefer to segregate in the approximate order: 10 > 9 > 8, and so on.

**Fig. 5:**
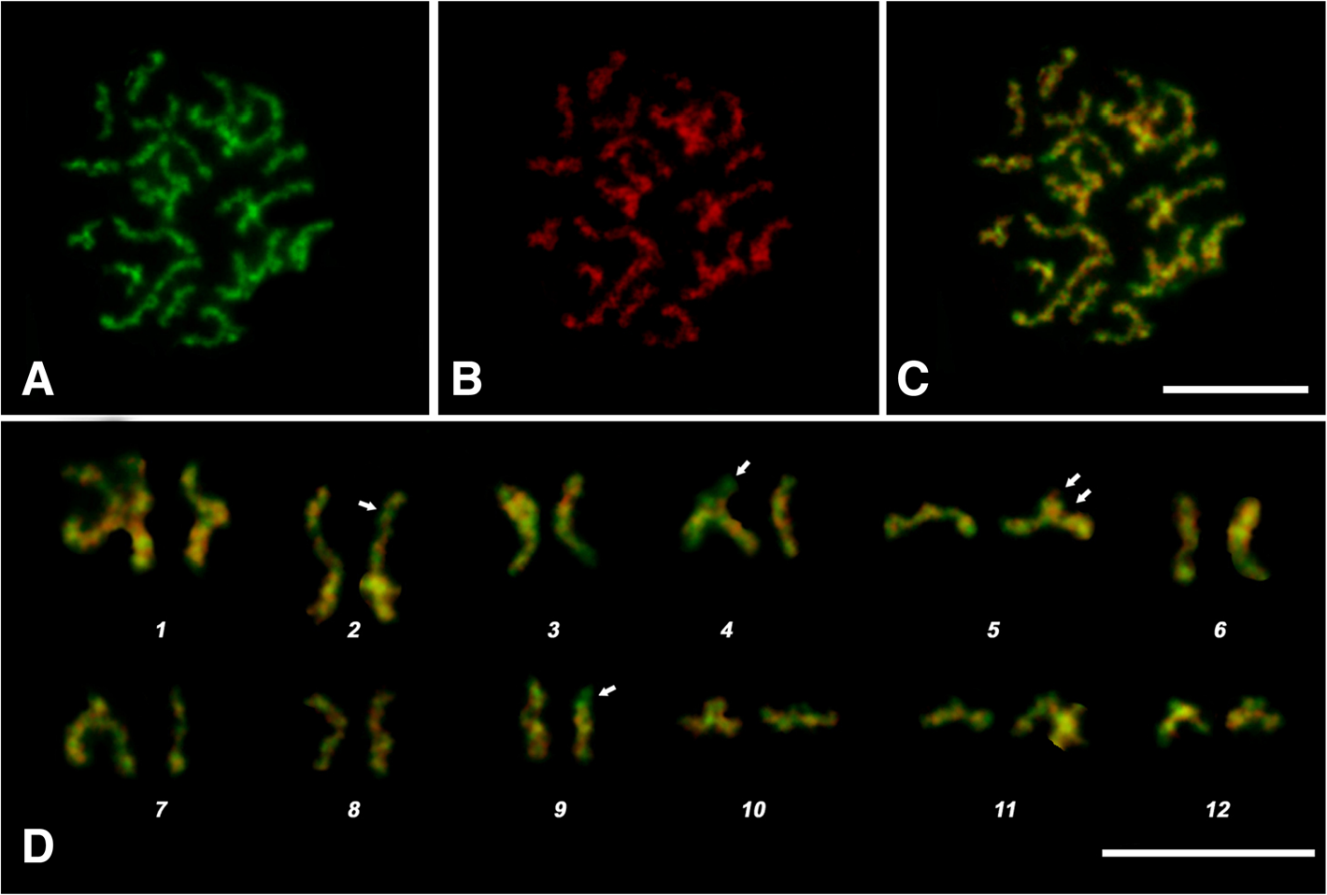
**a** *H. elongata* chromosomes stained by CMA_3_. **b** DAPI-stained chromosomes (**c**) merged image (**d**) karyotype derived from the cell shown in Fig. 6c. Arrows indicate polymorphic sites detected by double fluorochrome staining. Scale bar – 10 μm

Differential chromosome DAPI- and CMA_3_-staining revealed the chromatin areas that varied in AT- and GC-content. Both dyes stained the chromosomes all along but with fluctuating intensities in different areas. Staining differences were observed between homologues of several chromosomes (Fig. 5, Arrows). A number of terminal bands, dark with AT-specific fluorochrome DAPI, were stained quite pronouncedly by GC-specific fluorochrome CMA_3_, namely chromosomes 2, 4, 9 and 10. On the contrary, terminal and centromeric regions of chromosome 5 stained more intensive with DAPI than with CMA_3_ (Fig. 5).

There was variability in staining between homologs and sister chromatids. A clear example is seen on chromosome 4 (Fig. 5d). This kind of chromosome variability could be associated with the recombination that takes place during the sexual process between adult worms inside definitive host birds.

**Fig. 6:**
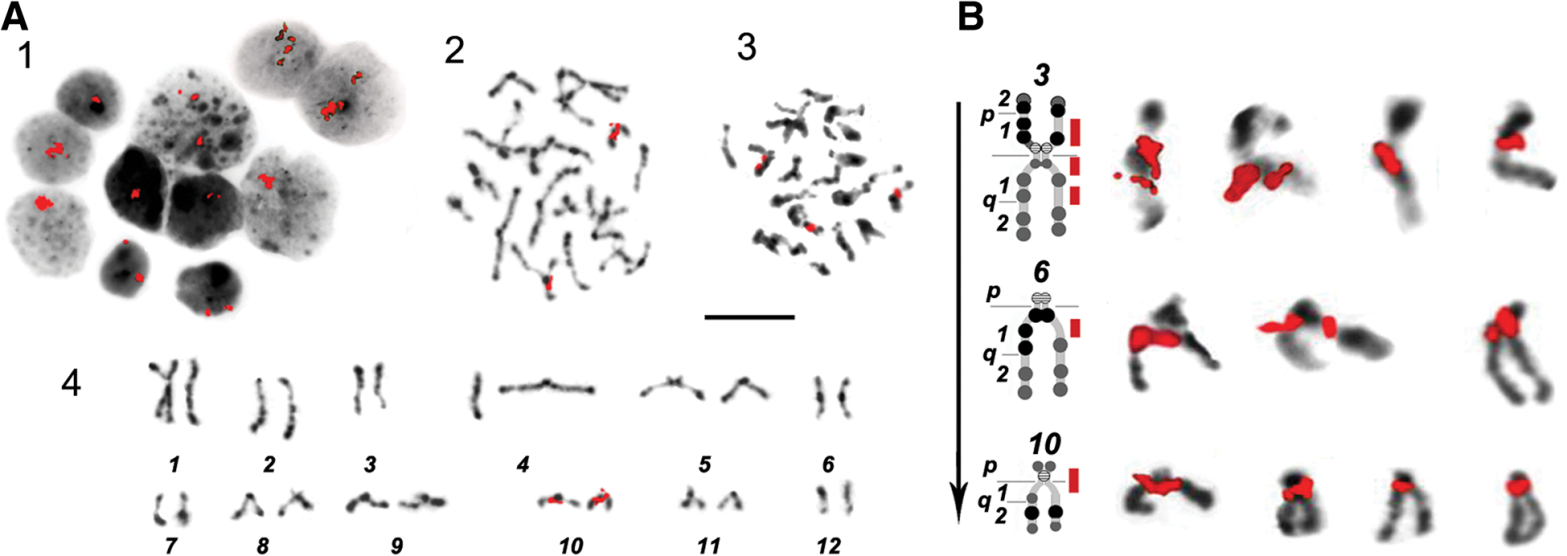
rDNA clusters revealed by FISH with 18S rDNA probe (red signals); chromosomes were counterstained with DAPI (in grey scale). **a** 1 – interphase nuclei. 2, 3 – premitotic and mitotic plates with rDNA signals. 4 – karyotype derived from the cell shown in (2). Scale bar – 10 μm. **b** 18S rDNA clusters on chromosomes 3,6,10. Chromosomes from different spreads are combined with their ideograms (Fig. 3). Black arrow indicates the increase of detected rDNA signals frequency

FISH of 18S rDNA probe revealed that the vast majority (95.0 %) of interphase nuclei contained one signal (Fig. 6 a, 1). One signal corresponds to the number of nucleoli per cell *in vivo* (Fig. 1). Sometimes one or two small nucleoli were also observed in addition to one large nucleolus (Fig. 6 a, 1). A maximum five signals were detected for 18S rDNA in interphase nuclei. That corresponded to NORs number, detected by Ag-staining (see Additional file 2: Supplementary methods and Additional file 3: Figure S2). The same was true for the chromosome spreads – most of them contained signal on two chromosomes. Three signals were detected in several cases. The rDNA clusters were located on chromosomes 3, 6 and 10. Physical mapping of 18S rDNA clusters on high-resolution *H. elongata* chromosomes (Fig. 6b) uncovered that chromosomes 3 and 10 could contain up to two loci simultaneously on the same arm or on the p and q arm of chromosomes. At the same time only one rDNA signal was detected in chromosome 6. Chromosome mapping of 18S rDNA in *H. elongata* (Fig. 6) revealed rDNA clusters in chromosome pairs 3, 6 and 10. Two labelled chromosome pairs were detected in 82.0 % of cells observed and three were singly labelled in 18.0 %. Only five combinations of two labeled chromosomes (of the six possible) per cell were seen, and a combination of chromosomes 3 and 6 was not observed. Approximately 78.9 % of evaluated spreads contained signals on chromosome 10 – 42.1 % with signal on pair 10 only, 26.3 % in combination with chromosome 3 and 10.5 % in combination with chromosome 6. Signals on chromosome 6 were found in 28.9 % of spreads (18.4 % with signal only on chromosome 6 and 10.5 % in combination with chromosome 10), and on chromosome 3 in 28.9 % of spreads (2.6 % of only chromosome 3 signal and 26.3 % in combination with chromosome 10). The size of signals at different chromosome pairs was generally equal. Ag-staining revealed 5 signals at chromosomes 3, 6 and 10 (Additional file 4: Figure S3). So at this stage of studies we suppose that chromosome 10 possesses the main rDNA cluster with the minor ones existing on chromosomes 3 and 6.

## Discussion

The goal of the work was the description of the *H. elongata* chromosome spreads, good enough for further cytogenetic and molecular researches.

In case of using conventional methods, metaphase cell accumulation observed only after prolonged 4–6 h of colchicine treatment of redia in culture medium and only clumpy metaphases unsuitable for future cytogenetic investigations obtained. It was impossible to determine more than one half of the metaphase chromosome set, while separate chromosomes were too condensed for bands identification. Up to 0.5 % per preparations of prometaphase cells suitable for band detection were obtained by suspending rediae after 4 h and 6 h of colchicine treatment. Thus, it was necessary to find the appropriate method for obtaining a larger amount of a better prometaphase or metaphase spreads. So *in vivo* observations helped to detect multiple dividing cells in several rediae tissues and cercarial embryos. High-pressure squash preparation used to improve chromosome spreads resolution. This delicate approach allows to get unbroken even fragile structures of polytene chromosomes. The results reproduced in multiple experiments at *H. elongata* spreads without significant chromosome damages.

There are morphologic polymorphisms in the number of spreads revealed varying levels of sister chromatid segregation (Tables 1 and 3) and discordance among homologous chromosomes. Partial sister chromatid segregation in *H. elongata* cells is always accompanied by a pronounced asymmetry in the level of chromatin condensation and (or) the band pattern. The phenomenon of homologue discordance is well established for human chromosomes. It was shown that the discordance minimum values (6.7 – 14.2 %) were observed at metaphase after GTG chromosome banding (G-banding with trypsin-Giemsa), whereas prometaphase chromosomes varied in size and had greater homolog discordance – up to 29.8 % after GTG and 64.2 % RBG (R-banding using bromodeoxyuridine and Giemsa) banding [32]. Homologue discordance has been widely discussed in the literature in terms of embryogenesis [33] and tissue specific stem cell differentiation [34], as well as asymmetry in cell division and tissue differentiation during embryogenesis [35–37]. Thus, the image bank for *H. elongata* karyotype component was made in order to detect all probable variabilities in chromosome morphology.

Representative rows for *H. elongata* individual chromosomes (Fig. 4) served for identification of individual chromosomes of prometaphase and metaphase sets. Individual chromosome variation was estimated by the following criteria: cohesion and incomplete sister chromatid segregation, banding pattern, centromeric heterochromatin and the possibility of distinguishing terminal bands by DAPI-staining. A more detailed description of homologs discordance and sister chromatids’ segregation variability requires additional study. Right now, we point out that one spread can contain homologs with resolutions corresponding to the extreme right and left positions in the chromosome identification rows (Fig. 5). The chromosome shapes are the superposition of two events: (1) the degree of condensation, which reflects mitosis advance; (2) the extent of sister chromatids’ segregation.

Centromeric heterochromatin blocks’ size variations, different from other morphological changes, may potentially reflect population variability. The source of such polymorphism could be found with the determination of species major tandem repeat, the main constituent of heterochromatic regions. It is possible that the first steps of *H. elongata* genome sequencing will bring up a major tandem repeat of this species. Such a repeat will serve as a reliable probe to assess heterochromatin block variability. Right now, it may only be supposed that possible heterozygosity of large heterochromatin blocks visible in many chromosome sets could be associated with cross fertilisation in adult flukes. The intercellular variation observed in heterochromatin does not exclude the possibility of polymorphism in parthenogenetic clonal populations.

Noticeable banding pattern along *H. elongata* chromosome arms visible after differential DAPI- and CMA_3_-staining. It is widely accepted that clear G- and R- blocks, typical for higher vertebrate chromosomes, are heterochromatic and euchromatic regions, respectively [38]. In the current work such banding is obtained for the flatworm karyotype for the first time. *Echinostoma caproni* is the nearest *H. elongata* relative, whose genome size is known – 0.85 pg [39]. Unfortunately, *E. caproni* karyotype is performed by Giemsa staining and C bands (centromeric) were described [10] and no detailed ideograms were made.

From 40.0 to 95.0 % of ribosomal DNA is in the inactive state in animal cells [40]. FISH identification of genes in heterochromatin requires increased chromosome deproteinisation to improve hybridization conditions. Chromosome mapping of inactive rDNA clusters is a common problem of animals cytogenetics with no universal solution so far. Even short-term treatment with pepsin and proteinase K led to the complete disappearance of the chromomere patterns on *H. elongata* chromosomes. We used the 18S rDNA FISH protocol without any protease treatment. All signals detected with rDNA FISH were also revealed by Ag-staining. Thus, there are at least 3 nucleoli organizing chromosomes in *H. elongata* karyotype.

Before very recent phylogenetic update [2] *H. elongata* belonged to Echinostomatidae Looss, 1899 family and following discussion focuses at “old” family state. Karyological data for the Echinostomatidae Looss, 1899 family was available for a rather small number of species whose intermediate hosts are freshwater molluscs and final hosts are birds, pets and humans. The diploid number of chromosomes in the Echinostomatidae family varies from 14 to 22 according to the literature available (Table 4). There is debate about the existence of an evolutionary tendency in the Digenea to maintain the diploid number equal to 20, which seems to be ancestral [11]. No karyological data on any of *Himasthla* species have been described yet. Based on chromosome number (2n = 24) *H. elongata* slightly deviates from the previously studied echinostomatids (2n = 14–22). *H. elongata* karyotype consists of two pairs of large chromosomes and 10 pairs of smaller-sized chromosomes. In contrast with a previous reports collected and analysed by Garcia-Souto and J. Pasantes [41] where single loci of major rDNA clusters are described in several digeneans, we revealed multiple signals of rDNA at 3 chromosome pairs in *H. elongata* karyotype. The lack of karyological data on the other *Himasthla* species doesn’t allow any speculation about chromosome number characteristic for this genus. More precise evaluation of karyotype components could be performed by geographic population of *H. elongata* expansion and involvement of other *Himasthla* species.

**Table 4:** Chromosome number described for Echinostomatidae Looss, 1899 family representatives before recent phylogenetic update [2]

Species	Chromosome number (2n)	Reference
*Echinochasmus baleocephalus*	14	Barsiene, Kiseliene, 1990 [42]
*Echinochasmus sp*	20	Staneviciute et al. 2015 [43]
*Echinoparyphium aconiatum*	20	Mutafova, Kanev, 1984 [44]
*Echinoparyphium recurvatum*	20	Mutafova et al. 1987 [45]
*Echinoparyphium pseudorecurvatum*	20	Barsiene et al. 1990 [46]
*Echinostoma barbosai*	22	Mutafova, Kanev, 1983 [47]
*Echinostoma caproni*	22	Richard, Voltz, 1987 [10]
*Echinostoma echinatum*	22	Mutafova, Kanev, 1983; 1986 [47, 48]
*Echinostoma revolutum*	22	Mutafova, Kanev, 1983; 1986 [47, 48]
*Echinostoma cinetorchis*	22	Terasaki et al. 1982 [49]
*Echinostoma hortense*	20	Terasaki et al. 1982 [49]
*Episthmium bursicola*	20	Barsiene, Kiseliene, 1990 [42]
*Hypoderaeum conoideum*	20	Mutafova et al. 1986 [50]
*Isthmiophora melis*	20	Mutafova, et al. 1991 [12]
*Moliniela anceps*	20	Barsiene et al. 1990 [51]
*Neoacantoparyphium echinoides*	20	Mutafova, 1994 [13]
*Paryphostomum radiatum*	16	Mutafova, 1994 [13]
*Pegosomum asperum*	20	Aleksandrova, Podgornova, 1978 [52]
*Pegosomum saginatum*	20	Aleksandrova, Podgornova, 1978 [52]
*Sphaeridiotrema globulus*	22	Mutafova et al. 2001 [53]

## Conclusions

In the current work karyotype of the first representative of *Himastla* genus determined. It is shown that *H. elongata* karyotype consists of two pairs of large chromosomes and 10 pairs of smaller-sized chromosomes. Differential DAPI- and CMA_3_-staining revealed the AT- and GC-rich chromosome bands. The identification of nucleoli organizing chromosomes was performed with 18S rDNA FISH and Ag-staining. The main rDNA cluster was observed on chromosome 10 with minor examples on chromosomes 3 and 6.

## Additional files

Additional file 1: Figure S1. Flow cytometry profile of 2C peaks for the species indicated. X axis - the relative pripidium iodide (PI) fluorescence intensity at FL3 channel; Y-axis, the number of events. (TIF 88 kb) Additional file 2: Supplementary methods. (DOC 16 kb) Additional file 3: Figure S2. Ag-staining of *H. elongata* nuclei. Nuclei with more than two Ag-NORs detected are shown in B, C, E, F, G. Scale bar – 10 µm. (TIF 4102 kb) Additional file 4: Figure S3. Correspondence of Ag-NOR-staining and rDNA FISH signals at aneuploid metaphase spread of *H. elongata*. A – metaphase spread. B – karyotype of the metaphase spread shown in A. Asterisks mark the correspondent chromosomes with rDNA FISH signals at (main text, Fig. 6). Scale bar – 10 µm. (TIF 481 kb)
