# Supplementary figures and images for: Karyotype features of trematode Himasthla elongata
Source: Mol Cytogenet. 2016 Apr 29;9:34. doi: 10.1186/s13039-016-0246-8 (PMC4850635; doi:10.1186/s13039-016-0246-8)

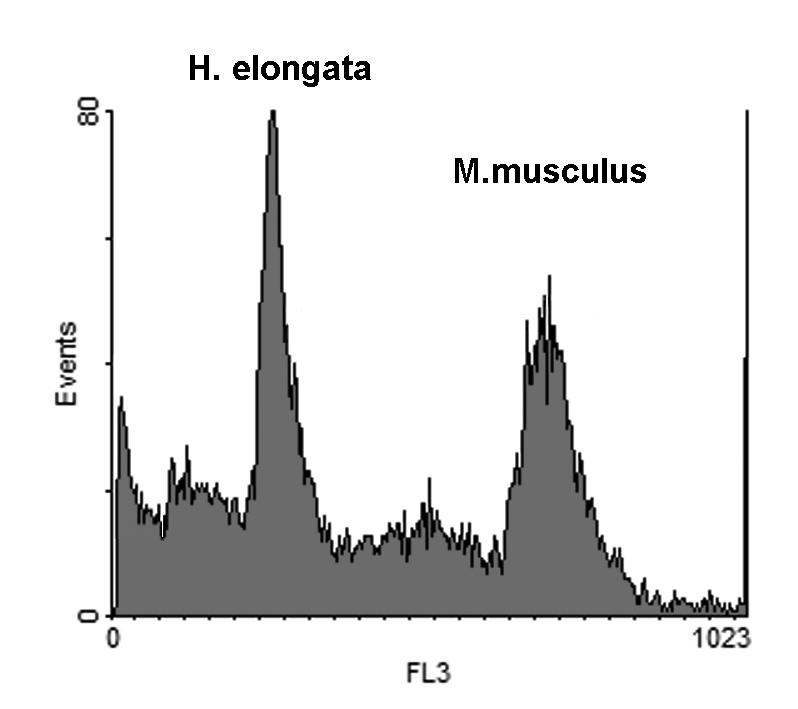

Supplement: Additional file 1: Figure S1. — Flow cytometry profile of 2C peaks for the species indicated. X axis - the relative pripidium iodide (PI) fluorescence intensity at FL3 channel; Y-axis, the number of events. (TIF 88 kb) [file 13039_2016_246_MOESM1_ESM.tif]

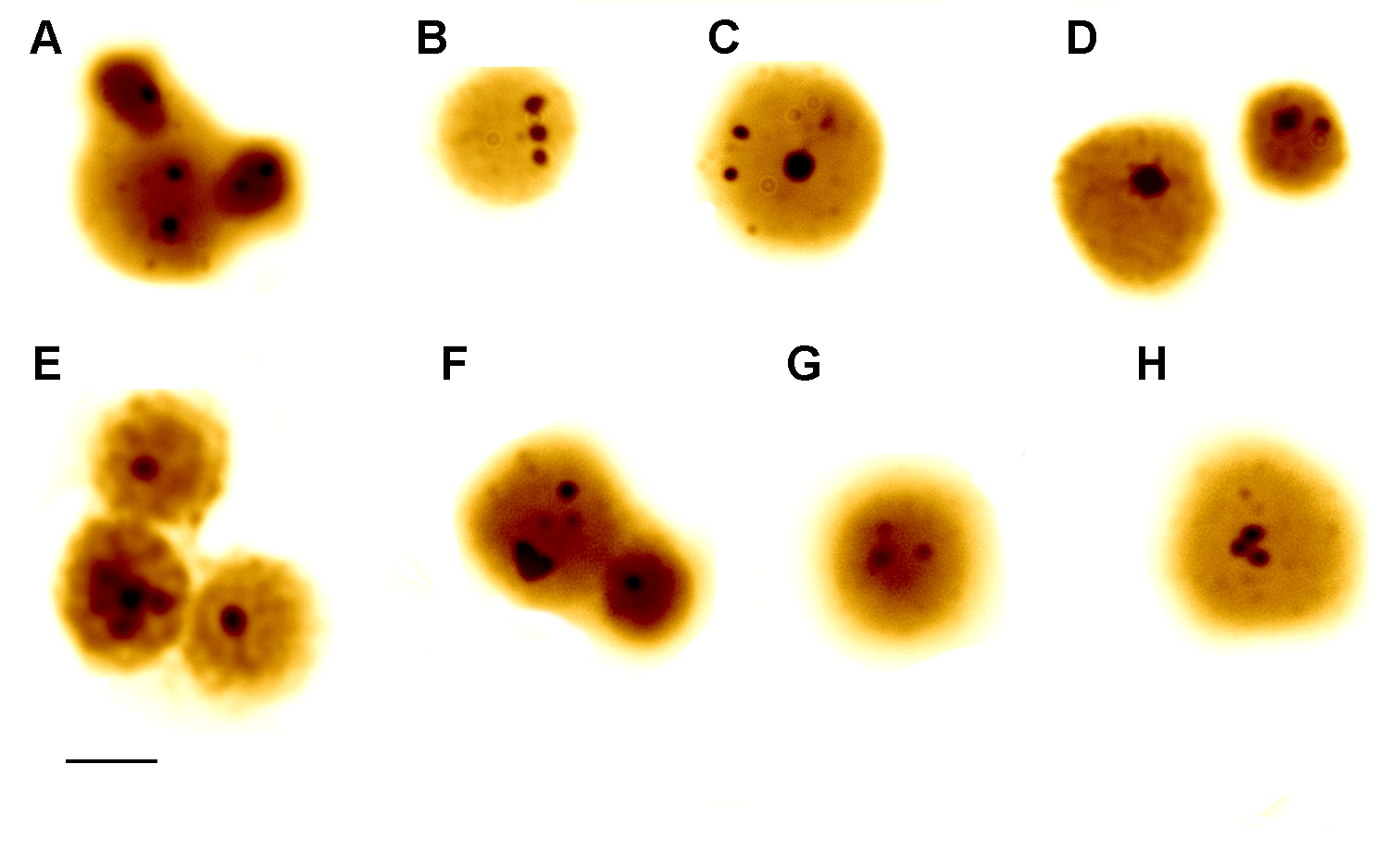

Supplement: Additional file 3: Figure S2. — Ag-staining of H. elongata nuclei. Nuclei with more than two Ag-NORs detected are shown in B, C, E, F, G. Scale bar – 10 µm. (TIF 4102 kb) [file 13039_2016_246_MOESM3_ESM.tif]

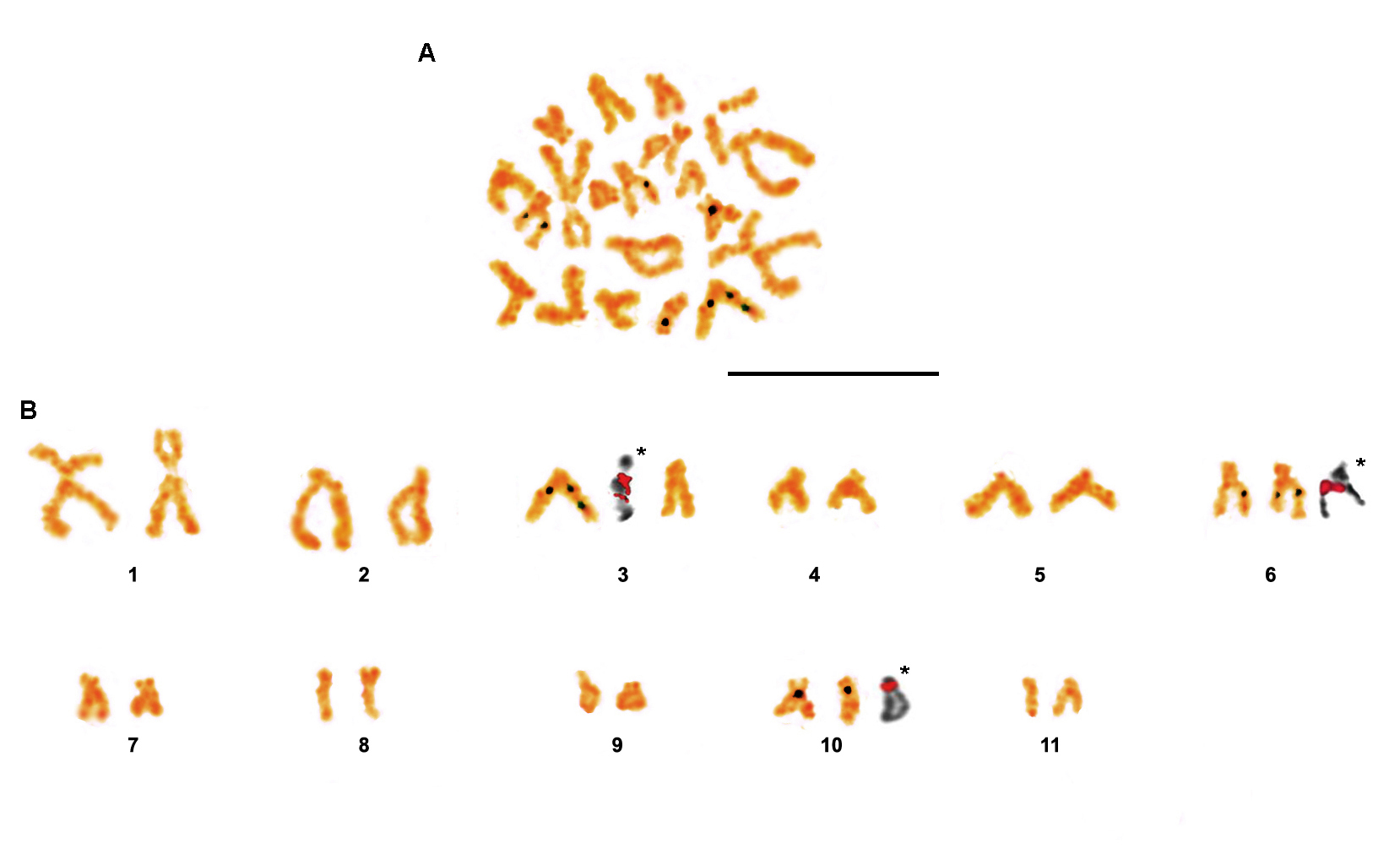

Supplement: Additional file 4: Figure S3. — Correspondence of Ag-NOR-staining and rDNA FISH signals at aneuploid metaphase spread of H. elongata. A – metaphase spread. B – karyotype of the metaphase spread shown in A. Asterisks mark the correspondent chromosomes with rDNA FISH signals at (main text, Fig. 6). Scale bar – 10 µm. (TIF 481 kb) [file 13039_2016_246_MOESM4_ESM.tif]
