# Supplementary material for: Karyotype features of trematode Himasthla elongata
Source: Mol Cytogenet. 2016 Apr 29;9:34. doi: 10.1186/s13039-016-0246-8 (PMC4850635; doi:10.1186/s13039-016-0246-8)
Supplement: Additional file 2: — Supplementary methods. (DOC 16 kb) [file 13039_2016_246_MOESM2_ESM.doc]

Supplementary methods

***H.elongata* nuclei isolation and flow cytometry**

Genome size was measured by flow cytometry. Mouse spleenocytes were used as the standard. Nuclei isolation and propidium iodide (PI) staining was modified from procedure published (Hare and Johnston, 2011). Whole rediae and murine spleen were homogenised separately in solution, containing 1х PBS, 0.05M EDTA, 0.01% Tween 20, 0.05M MgCl_2_, 7% glycerol and 0.5 mM PMSF (phenylmethylsulfonyl fluoride). Then nuclear suspensions were filtered ground through 40-mm nylon mesh, centrifuged at 2.5 krpm for 10 min and dissolved in 1x PBS with 10mM glycine, PI 50 and 100 µg/ml RNAse A to 1 million nuclei per 1 ml, mixed together and incubated for 30 minutes at +4 °C. Flow cytometry of PI stained nuclei was carried out at Epics XL (Beckman Coulter, USA) flow cytometer 488 nm(FL3) extraction path. Measurements were repeated 5 times, at least ten thousand nuclei of both species were taken in account for genome size determination.

**NOR silver staining method**

Ag-stainig was performed according to protocol published (Howell and Black, 1980). Nuclei and chromosome preparations after high-pressure treatment were used for AgNORs detection. One drop of gelatin solution and two drops of silver nitrate solution (50%) were placed on slide, gently mixed and covered with cover glass. The slide was placed in a wet chamber at 65-70°C until the solution turns golden brown. The slide was rinsed in several changes of distilled water and dried. Gelatin solution are prepared by dissolving 2g of gelatin and 1 ml of formic acid in 100 ml of distilled water.

Supplementary references

# Hare E, Johnston J. Genome size determination using flow cytometry of propidium iodide-stained nuclei. Methods Mol Biol., 2011; 772 : 3 – 12.

Howell WM, Black DA. Controlled silver-staining of nucleolus organizer regions with a protective colloidal developer: a 1-step method. Experientia. 1980; 36(8): 1014 – 5.
